# Pharmacometabolomic Pathway Response of Effective Anticancer Agents on Different Diets in Rats with Induced Mammary Tumors

**DOI:** 10.3390/metabo9070149

**Published:** 2019-07-22

**Authors:** Zhijun Cao, Mark Steven Miller, Ronald A. Lubet, Clinton J. Grubbs, Richard D. Beger

**Affiliations:** 1 Division of Systems Biology, National Center for Toxicological Research, Jefferson, AR 72079, USA; 2 Chemopreventive Agent Development Research Group, Division of Cancer Prevention, National Cancer Institute, Bethesda, MD 20892, USA; 3 Department of Surgery, Chemoprevention Center, University of Alabama at Birmingham, Birmingham, AL 35233, USA

**Keywords:** pharmacometabolomics, standard diet, high fat diet, protective anticancer agent, targretin, tamoxifen

## Abstract

Metabolomics is an effective approach to characterize the metabotype which can reflect the influence of genetics, physiological status, and environmental factors such as drug intakes, diet. Diet may change the chemopreventive efficacy of given agents due to the altered physiological status of the subject. Here, metabolomics response to a chemopreventive agent targretin or tamoxifen, in rats with methylnitrosourea-induced tumors on a standard diet (4% fat, CD) or a high fat diet (21% fat, HFD) was evaluated, and found that (1) the metabolome was substantially affected by diet and/or drug treatment; (2) multiple metabolites were identified as potential pharmacodynamic biomarkers related to targretin or tamoxifen regardless of diet and time; and (3) the primary bile acid pathway was significantly affected by targretin treatment in rats on both diets, and the lysolipid pathway was significantly affected by tamoxifen treatment in rats on the high fat diet.

## 1. Introduction

The rat model of dimethylbenzanthracene or methylnitrosourea (MNU) induced breast cancer has been employed in the development of new chemopreventive agents, which is highly responsive to a number of regimens that are both hormone related (selective estrogen receptor modulators (SERM), aromatase inhibitors, ovariectomy) [1,2,3] and non-hormone related (agonists for the retinoid X receptor) [4,5]. Metformin, an antidiabetic drug, has gained attention both in cancer prevention and therapy, since the epidemiologic data supported that metformin may reduce cancer risk in diabetic patients [6]. However, our study found that metformin was completely ineffective in the rat MNU model [7]. Diet has been found to affect chemical induced cancer in rats [8,9]. These MNU rat model studies have been performed with rats on a standard diet with a relatively low level of fat using soy as a protein source. In contrast, the standard human diet in the United State has high levels of fat. The altered diet might change physiologic status or pharmacokinetics, resulting in a change in the efficacy of chemopreventive agents. As such, it is necessary to address the influences of diet on efficacy of chemopreventive agents in the MNU rat model.

In our previous studies, the Western diet enhanced tumor formation in the ER+ rat mammary model while the response to the various agents was the same with both diets. Thus, targretin (Tgt), tamoxifen (Tmx), and vorozole (aromatase inhibitor) were all clearly and similarly positive in rats on either diet whereas metformin and Lipitor gave negative results on either diet. We employed the MNU-rat model because it is perhaps the most standard ER+ model of breast cancer and was employed in the development of SERMs (tamoxifen, raloxifene, toremifene) and aromatase inhibitors (letrozole, vorozole), which are indeed the standard prevention and treatment modalities for ER+ breast cancer in humans [10]. The study was initially undertaken to determine whether the Western Diet, which might be considered a “more relevant” diet for comparison to humans, gave similar results with various prevention/therapeutic agents. The metabolomics was also performed to identify potential pharmacodynamic biomarker candidates with various agents [10].

Here, we performed a complete pattern analysis of metabolomics data and examined metabolomic and pathway changes that were associated with response to different diets, and two effective anticancer agents Tgt or Tmx in MNU-induced cancer rats fed with a standard diet (4% fat, CD) or a high fat diet (21% fat, HFD).

## 2. Results

**Figure 1:**
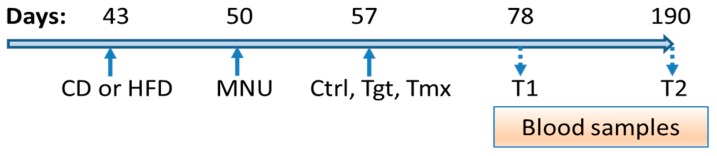
Diet and Treatment process. Diets: standard diet (CD); high fat diet (HFD); Times: T1, 78 days of age; T2, 190 days of age; Treatments: Ctrl, control; Tgt, targretin; Tmx, tamoxifen.

Figure 1 shows the timeline for administration of diet, MNU, and preventive agents. On day 43, Sprague-Dawley rats were started on CD or HFD diet. On day 50, MNU was given to initiate tumorgenesis. On day 57, either control vehicle, or the preventive agents Tgt or Tmx, were started and continued until the end of the study. Blood samples were obtained at day 78 (T1) and day 190 (T2) for both control and treated rats; metabolomic analysis of those samples was done at Metabolon as previously described [10].

### 2.1. Principal Component Analysis (PCA) and Partial Least Square Discriminant Analysis (PLS-DA)

**Figure 2:**
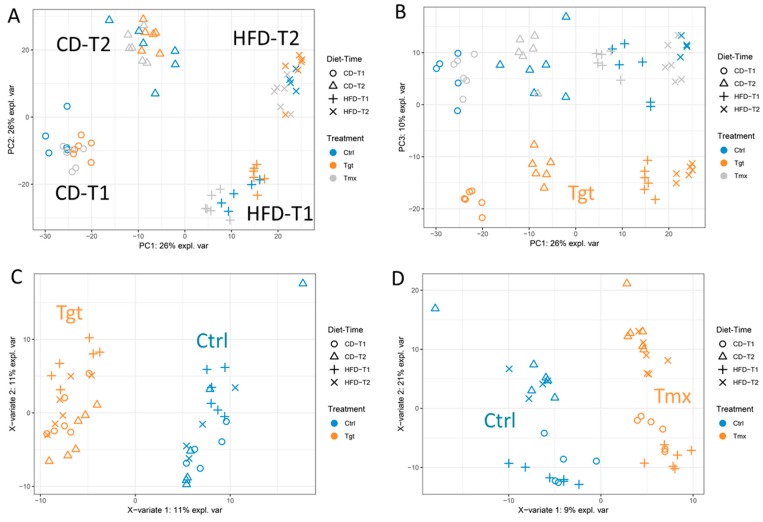
Multivariate analysis of all quantified metabolites. (**A**,**B**) the scores plots of the PCA analysis, (**C**,**D**) the scores plots of the PLS-DA analysis. Diets: standard diet (CD); high fat diet (HFD); Times: T1, 78 days of age; T2, 190 days of age; Treatments: Ctrl, control; Tgt, targretin; Tmx, tamoxifen.

In total 565 metabolites were semi-quantified (Table S1). PCA is an unsupervised multivariate analysis to reduce the complexity of data and reveal the internal structure of the data. PCA analysis were performed on semi-quantified 565 metabolites with log-transformation to explore the effect of time, diet, and drug treatment. As shown in the scores plot of the PCA Figure 2A, samples are clustered into four groups based on diets and time points: control diet at time point 1 and 2 (CD-T1, CD-T2), high fat diet at time point 1 and 2 (HFD-T1, HFD-T2). The first component (PC1) mainly describes the difference between diets; the second component (PC2) mainly describes the difference between time points. Interestingly, as shown in the scores plot of components 1 and 3 Figure 2B, Tgt treated samples are well separated from control and Tmx treated groups.

Regarding the effect of drug treatment on the metabolome, a supervised multivariate analysis PLS-DA was employed for each treatment and control. As shown in the scores plots of PLS-DA Figure 2C,D, samples are classified into treatment (Tgt or Tmx) and Ctrl. The first component mainly describes the difference between the treatment group and the control group.

In order to discover potential pharmacodynamic markers for the drug treatment, the metabolites with the highest contribution for group separations are shown in Figure S1. The top 10 metabolites contributing to the Tgt group for the first component are stearoyl-arachidonoyl-glycerophosphoethanolamine (1)*, citrulline, palmitoyl-arachidonoyl-glycerophosphocholine (2)*, targretin, palmitoyl-oleoyl-glycerophosphocholine (1)*, phenylacetylglycine, stearoyl sphingomyelin, oleoyl-linoleoyl-glycerophosphocholine (1)*, 3-methylcrotonylglycine, and nervonoyl sphingomyelin*. These metabolites are involved subpathway including lysolipid, urea cycle, drug, phenylalanine and tyrosine metabolism, sphingolipid metabolism, etc.

The top 10 metabolites contributing to the Tmx group for the first component are oleoyl-linoleoyl-glycerophosphoinositol (1)*, 1-palmitoylglycerophosphoinositol*, palmitoyl-linoleoyl-glycerophosphocholine (2)*, palmitoyl-linoleoyl-glycerophosphocholine (1)*, palmitoyl-linoleoyl-glycerophosphoinositol (1)*, 1-oleoylglycerophosphoinositol*, 1-palmitoylglycerophosphoethanolamine, 1-palmitoylglycerophosphate, glycohyodeoxycholate, and heme. Interestingly, eight of them are involved in the lysolipid sub-pathway.

### 2.2. Differentially Regulated Metabolites

**Figure 3:**
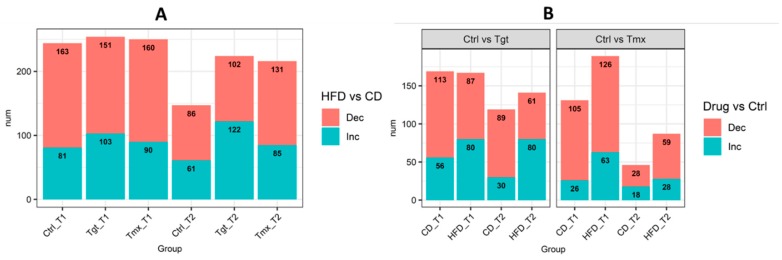
(**A**) Number of metabolites significantly changed in abundance in HFD vs. CD for each group; (**B**) Number of metabolites significantly changed in abundance in Drug vs. Ctrl for each group. Pink shows metabolites decreased and blue shows metabolites increased. Diets: standard diet (CD); high fat diet (HFD) Times: T1, 78 days of age; T2, 190 days of age; Treatments: Ctrl, control; Tgt, targretin; Tmx, tamoxifen.

Metabolite levels were compared between the CD/HFD groups and control/drug treatment groups at time points 1 and 2 (Table S2) with Welch two-sample t test. Metabolite level change with *p* < 0.05, FDR < 0.2, and fold change ≥1.2 was considered as a statistically significantly change. As shown in Figure 3A, diet can significantly affect the metabolome. In total, there are 244, 254, and 250 metabolites significantly altered in abundance at time point 1, and 147, 224, and 216 metabolites altered in abundance at time point 2 for control, Tgt and Tmx. At T2, more metabolites were altered in abundance in the drug treatment groups (Tgt and Tmx) than control. There are more metabolites significantly changed in abundance at time point 1 than at time point 2, and the presence of Tgt or Tmx resulted in additional metabolites changes in abundance that were related to the difference in diets at time point 2.

As shown in Figure 3B, Tgt treatment resulted in 169 and 167 metabolites significantly altered in abundance at time point 1 and 119 and 141 metabolites significantly altered in abundance at time point 2 for CD and HFD, respectively. Tmx treatment resulted in 131 and 189 metabolites significantly altered in abundance at time point 1 and 46 and 87 metabolites significantly altered in abundance at time point 2 for CD and HFD, respectively. There are more metabolites significantly altered in abundance at time point 1 than at time point 2 for both drugs regardless of the diet. Tgt treatment resulted in more metabolites significantly altered in abundance for HFD than for CD at time point 2, while Tmx treatment resulted in more metabolites significantly altered in abundance for HFD than for CD at both time points.

**Figure 4:**
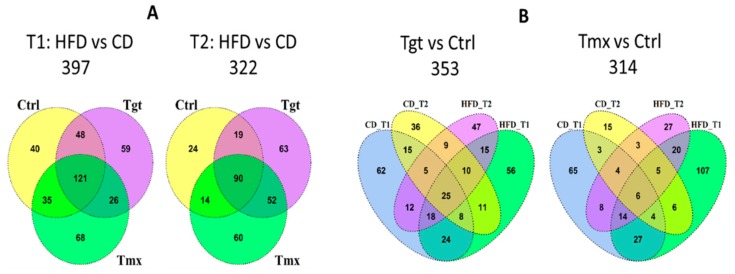
Venn diagrams of metabolites significantly changed in abundance among groups. (**A**) overlap of metabolites significantly changed in abundance at T1 and T2 for HFD vs. CD; (**B**) overlap of metabolites significantly changed in abundance for Tgt vs. control and Tmx vs. Ctrl. Diets: standard diet (CD); high fat diet (HFD); Times: T1, 78 days of age; T2, 190 days of age; Treatments: Ctrl, control; Tgt, targretin; Tmx, tamoxifen.

**Figure 5:**
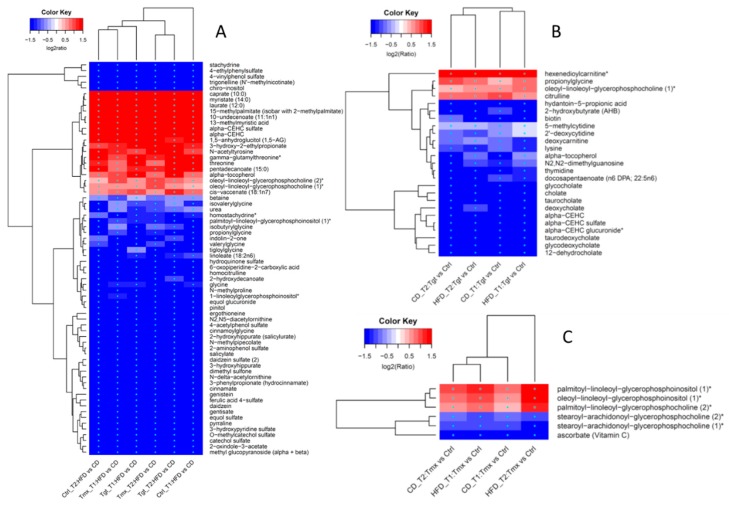
Hierarchical clustering analysis heatmaps of fold changes on the log2 scales. (**A**) Diet associated (HFD vs. CD); (**B**) Tgt associated (Tgt vs. Ctrl); (**C**) Tmx associated (Tmx vs. Ctrl). * indicates *p* value < 0.05, FDR < 0.2, and fold change ≥1.2. Diets: standard diet (CD); high fat diet (HFD); Times: T1, 78 days of age; T2, 190 days of age; Treatments: Ctrl, control; Tgt, targretin; Tmx, tamoxifen.

As shown in Figure 4A, a total of 397 and 322 metabolites are significantly altered in abundance at T1 and T2 when comparing HFD to CD, respectively, among which 121 and 90 metabolites are common among treatment groups (Ctrl, Tgt, and Tmx) at T1 and T2, respectively. Moreover, 68 metabolites are common between those 121 and 90 metabolites. Figure 5A shows the heatmap of fold change for those 68 metabolites, 18 out of 68 metabolites involved in the sub-pathways of long/medium chain fatty acid, fatty acid branched, tocopherol metabolism, etc., exhibit significantly higher levels in the HFD than in the CD group, while the rest of the 50 metabolites involved in the sub-pathways of food component/plant, benzoate metabolism, urea cycle, etc., exhibit significantly lower levels in the HFD than the CD group, regardless of the presence or absence of Tgt or Tmx and the time points examined.

As shown in Figure 4B (left), Tgt treatment resulted in 25 metabolites significantly altered in abundance which are common to both diets at both time points. As shown in Figure 5B, four of the 25 metabolites-hexenedioylcarnitine*, propionylglycine, oleoyl-linoleoyl-glycerophosphocholine (1)*, and citrulline are related to fatty acid metabolism, lysolipid, and the urea cycle pathways and are significantly increased when comparing Tgt treatment to Ctrl. In contrast, 21 out of the 25 metabolites related to primary/secondary bile acid metabolism (taurocholate, cholate, glycocholate, deoxycholate, taurodeoxycholate, glycodeoxycholate, 12-dehydrocholate), tocopherol metabolism (alpha-tocopherol, alpha-CEHC, alpha-CEHC sulfate, alpha-CEHC glucuronide*), pyrimidine metabolism (2’-deoxycytidine, 5-methylcytidine, thymidine), etc., are significantly decreased in the Tgt group vs. Ctrl.

As shown in Figure 4B (right), Tmx treatment resulted in 6 metabolites significantly altered in abundance which are common to CD and HFD at both time points. As shown in Figure 5C, 3 of the 6 metabolites -oleoyl-linoleoyl-glycerophosphoinositol (1)*, palmitoyl-linoleoyl-glycerophosphoinositol (1)*, palmitoyl-linoleoyl-glycerophosphocholine (2)*—which are related to the lysolipid pathway were significantly increased from Tmx treatment vs. Ctrl. The rest of the three metabolites ascorbate, stearoyl-arachidonoyl-glycerophosphocholine (2)*, and stearoyl-arachidonoyl-glycerophosphocholine (1)*—which are related to ascorbate and aldarate metabolism and lysolipid pathways are significantly decreased by Tmx treatment compared to the control.

### 2.3. Pathway Enrichment Analysis

**Figure 6:**
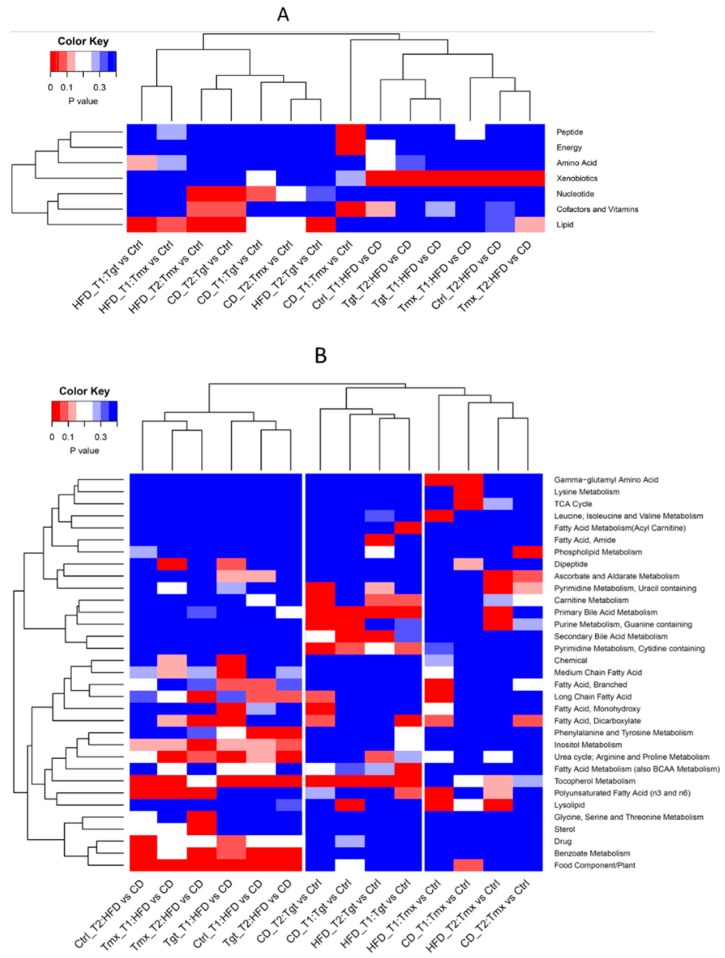
Hierarchical clustering analysis heatmaps of *p* values of pathway enrichment using Fisher’s exact test. (**A**) super pathways; (**B**) sub-pathways. Diets: standard diet (CD); high fat diet (HFD); Times: T1, 78 days of age; T2, 190 days of age; Treatments: Ctrl, control; Tgt, targretin; Tmx, tamoxifen.

Super pathway enrichment: As shown in Figure 6A in the hierarchical clustering analysis (HCA) heatmap, the xenobiotics pathway is significantly changed by diets (HFD vs. CD). Lipid, cofactors and vitamins, nucleotide, energy, and peptide pathways are significantly changed by either Tgt or Tmx treatments.

**Figure 7:**
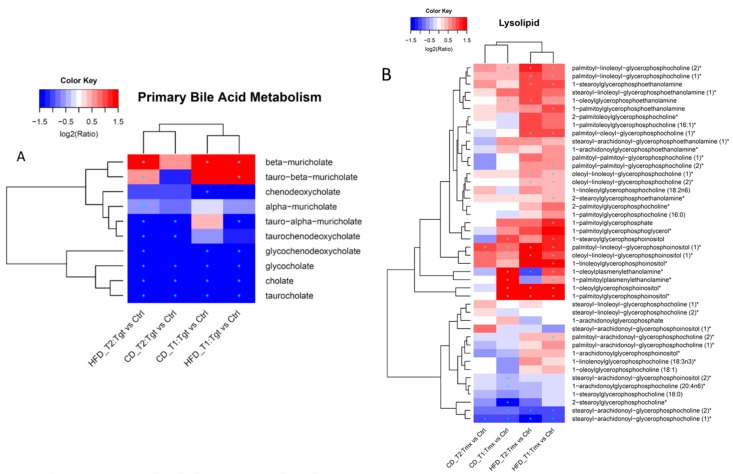
Hierarchical clustering analysis heatmaps of fold changes on the log2 scales. (**A**) metabolites in sub pathway primary bile acid metabolism related to Tgt treatment; (**B**) metabolites in sub pathway lysolipid related to Tmx treatment. * indicates *p* value < 0.05, FDR < 0.2, and fold change ≥1.2. Diets: standard diet (CD); high fat diet (HFD); Times: T1, 78 days of age; T2, 190 days of age; Treatments: Ctrl, control; Tgt, targretin; Tmx, tamoxifen.

Sub pathway enrichment: As shown in Figure 6B, many sub-pathways are affected by diet and/or drug treatment. Interestingly, based on the *p* value of sub pathway enrichment analysis, comparison of sample groups analysis demonstrate segregation into two clusters: diet associated cluster (HFD vs. CD) and protective anticancer agents associated cluster, which can be further clustered into Tgt treatment cluster (Tgt vs. Ctrl), and Tmx treatment cluster (Tmx vs. Ctrl). Changes of the food component/plant, benzoate, metabolism, urea cycle, etc., are associated with diets; changes of primary/secondary bile acid metabolism, lysolipid, pyrimidine metabolism, etc., are associated with Tgt and/or Tmx treatment. Specific metabolite changes from pathways of interest including primary bile acid metabolism, lysolipid pathways are shown in Figure 7.

## 3. Discussion

Our previous article demonstrated that diets affected the development of MNU-induced breast cancer in rats, where the HFD resulted in increased tumor multiplicity and tumor weight and decreased tumor latency compared to CD. Anticancer protection agents, Tgt and Tmx, yielded strong positive results (reduced tumor multiplicity and weights, as well as increased tumor latency) not only in the rats on CD but also in rats on HFD. Serum metabolites associated with diets and Tgt and Tmx treatments were also examined briefly by metabolomics analysis [10]. Here, we systematically reviewed metabolic changes related to diets and drug treatments with the aim to identify specific metabolites as biomarkers for the physiological or pharmacological effect of a given agent at an effective preventive dose in the animal model.

PCA analysis found that diets and Tgt treatment had substantial effects on changes of the serum metabolome in rats at both time points. Interestingly, the effects of either Tgt or Tmx treatment on rat serum metabolome were clearly revealed by PLS-DA analysis Figure 2C,D; the metabolites which had high contributions to the outcomes may be of interest as potential pharmacodynamic biomarker candidates for the drug treatment (Figure S1).

Regardless of time points or drugs, there were 68 metabolites altered in abundance consistently by feeding different diets (Figure 5A), and the altered metabolites may be directly related to the composition of the diets. For example, higher levels of some long/medium chain fatty acid, branched fatty acids in rats fed with HFD might be related to the high fat content in the HFD, while rats fed with CD had higher levels of genistein, daidzein, and equol might be related to the high soy content in the CD [10]. This is consistent with the results of the pathway analysis which showed that the food component/plant pathway was altered due to the different diets.

Metabolites that were significantly altered in abundance by Tgt or Tmx treatment at both time points and both diets (CD and HFD) are shown in Figure 5B,C respectively, so these metabolites or metabolic patterns could be potential pharmacodynamic biomarker candidates.

Drug treatments caused changes of a few sub-pathways in the rats fed with CD and/or HFD at T1 and/or T2 (see Figure 6B). One of the pathways of interest is the primary bile acid pathway, which was enriched by Tgt treatment in rats given either CD or HFD at both time points. As shown in Figure 7A, seven metabolites were decreased and one (beta-muricholate) was increased at both time points and both diets during Tgt treatment. The changes in the bile acids are in line with our prior study where Tgt, a retinoid X receptor agonist, altered bile acids in liver tissue [11], while glycochenodeoxycholate and taurochenodeoxycholate had opposite responses in the two studies. This difference may be due to different sample types and sample collection time. Boxplots for these changed metabolites are shown in Figure S2.

In Figure 6B, another sub pathway of interest is the lysolipid sub pathway, which was significantly affected by Tmx for HFD at both time points. Figure 7B provides a heatmap of the detailed metabolites related sub-pathway lysolipid for Tmx response. The increase of palmitoyl-linoleoyl-glycerophosphocholine (2)* and the decrease of stearoyl-arachidonoyl-glycerophosphocholine (1)* resulted from Tmx treatment are consistent with the previously reported findings [12]. Oleoyl-linoleoyl-glycerophosphocholine (1)*, oleoyl-linoleoyl-glycerophosphocholine (2)*, palmitoyl-oleoyl-glycerophosphocholine (1)*, palmitoyl-linoleoyl-glycerophosphocholine (1)*, and 1-stearoylglycerophosphoethanolamine were significantly increased in rats on HFD, not CD; 1-stearoylglycerophosphoinositol, and 1-palmitoylplasmenylethanolamine* were significantly increased in rats at T1 not T2. These metabolites may be considered as diet and time sensitive pharmacodynamic biomarker candidates.

## 4. Materials and Methods

**Chemicals**. Targretin was supplied by the NCI Cancer Prevention Repository (Bethesda, MD, USA). Tamoxifen citrate was obtained from Sigma Chemical Co. (St. Louis, MO, USA). Other agents were incorporated into the diet as described previously [2,13].

**Chemoprevention studies**. Treatment of female Sprague–Dawley rats was conducted as described previously [3,14]. In brief, rats (obtained from Envigo, Inc., Huntingdon, CBG, UK) were housed in Institutional Animal Care and Use Committee (IACUC)-approved animal facilities at the University of Alabama at Birmingham (Birmingham, AL, USA) beginning at 35 days of age (DOA). Rats were placed on a standard Teklad 7001 diet (4% fat, high calcium and high soy protein) or a Western diet (HFD), Envigo TD 88,137 (21% fat, low in calcium, and no soy proteins) at 43 DOA. MNU (99.5% pure) was obtained from the NCI and was injected intravenously [75 mg/kg body weight (BW)] via the jugular vein when the animals were 50 DOA. At 57 days of age, animal treatments with the various agents were initiated daily and continued throughout the duration of the study. Doses were 3.3 and 150 ppm in diet for tamoxifen and targretin, respectively. Serum was obtained from rats at both 78 days and at the time of sacrifice (190 DOA).

**Metabolic profiling**. The methods employed were described in our previous publication [11]. The nontargeted metabolic profiling platform employed three independent platforms: ultra–high-performance LC/MS-MS (UHPLC/MS/MS) optimized for either basic or acidic species, and gas chromatography/mass spectrometry (GC/MS). Samples were processed as described previously [15,16]. For each sample, serum protein was precipitated with methanol. The supernatant was split equally for analysis on the three platforms. Aliquots, dried under nitrogen and vacuum-desiccated, were subsequently either reconstituted in 50 µL 0.1% formic acid in water (acidic conditions) or in 50 µL 6.5 mmol/L ammonium bicarbonate in water, pH 8 (basic conditions) for the two UHPLC/MS/MS analyses or derivatized to a final volume of 50 µL for GC/MS analysis using equal parts bistrimethylsilyl-trifluoroacetamide and solvent mixture acetonitrile: dichloromethane: cyclohexane (5:4:1) with 5% triethylamine at 60 °C for 1 h. A pooled sample was generated by taking a small volume from each sample and was used as a technical replicate throughout the data set. Extracted water samples served as process blanks. A proprietary cocktail of quality control standards that were carefully chosen not to interfere with the measurement of endogenous compounds was spiked into every analyzed sample and was used to monitor instrument performance and chromatography alignment. Instrument variability was determined by calculating the median relative standard deviation (RSD) for the standards that were added to each sample prior to injection into the mass spectrometers. Overall process variability was determined by calculating the median RSD for all endogenous metabolites (i.e., non-instrument standards) present in 100% of the pooled matrix samples. Experimental samples were randomized across the platform run with QC samples spaced evenly among the injections.

Briefly, for UHPLC/MS/MS analysis, the metabolites from the serum-extracted aliquots were separated using a Waters Acquity UPLC (Waters, Milford, MA, USA) system. Data was collected using an LTQ mass spectrometer (Thermo Fisher Scientific, Waltham, MA, USA), which consisted of an electrospray ionization (ESI) source and linear ion-trap (LIT) mass analyzer. The MS instrument scanned 99–1000 m/z and alternated between MS and MS2 scans using dynamic exclusion with approximately 6 scans per second. For GC/MS analysis, the derivatized samples were separated on a 5% phenyldimethyl-silicone column with helium as the carrier gas and a temperature ramp from 60 °C to 340 °C and then analyzed on a Thermo-Finnigan Trace DSQ MS (ThermoFisher Scientific, Waltham, MA, USA) operated at unit mass resolving power with electron impact ionization and a 50–750 atomic mass unit scan range.

Metabolites were identified by automated comparison of the ion features in the experimental samples to an in-house reference library of chemical standard entries that included retention time, molecular weight (m/z), preferred adducts, and in-source fragments as well as associated MS spectra, and curated for quality control using software [17]. For statistical analyses, any missing values were assumed to be below the limit of detection and these values were imputed with the compound minimum (minimum value imputation). Principal component analysis and statistical analysis of the log-transformed data were performed using a free open source software package “R” (http://cran.r-project.org/). A Welch two-sample t-test was used for statistical analysis. The Benjamini and Hochberg method was used to control the false discovery rate (FDR) across biochemicals [18]. Metabolites with *p* < 0.05, FDR < 0.2, and fold change ≥1.2 were considered statistically significant. Pathway enrichment analysis was done by Fisher’s exact test.

## 5. Conclusions

In summary, we systematically reviewed metabolic changes related to diets and drug treatments at different time points in rats with MNU induced breast cancer and found that (1) the metabolome was substantially affected by diet and/or drug treatment; (2) multiple metabolites were identified as potential pharmacodynamic biomarkers related Tgt or Tmx regardless diets and time; and (3) primary bile acid pathway was significantly affected by Tgt treatment in rats on both diets, and lysolipid pathway was significantly affected by Tmx treatment rats on the high fat diet.

## Supplementary Materials

The following are available online at https://www.mdpi.com/2218-1989/9/7/149/s1, Figure S1. contribution of top 120 metabolites for PLS-DA, Figure S2. Boxplots for metabolites changed in primary bile pathway. Diets: CD, standard diet; HFD, high fat diet; time: T1, 78 days of age; T2, 190 days of age; treatment: Ctrl, control; Tgt, targretin; Tmx, tamoxifen. Table S1. quantified metabolites, Table S2. statistical analysis results.
