# Supplementary material for: Pharmacometabolomic Pathway Response of Effective Anticancer Agents on Different Diets in Rats with Induced Mammary Tumors
Source: Metabolites. 2019 Jul 22;9(7):149. doi: 10.3390/metabo9070149 (PMC6680681; doi:10.3390/metabo9070149)

## Slide 1
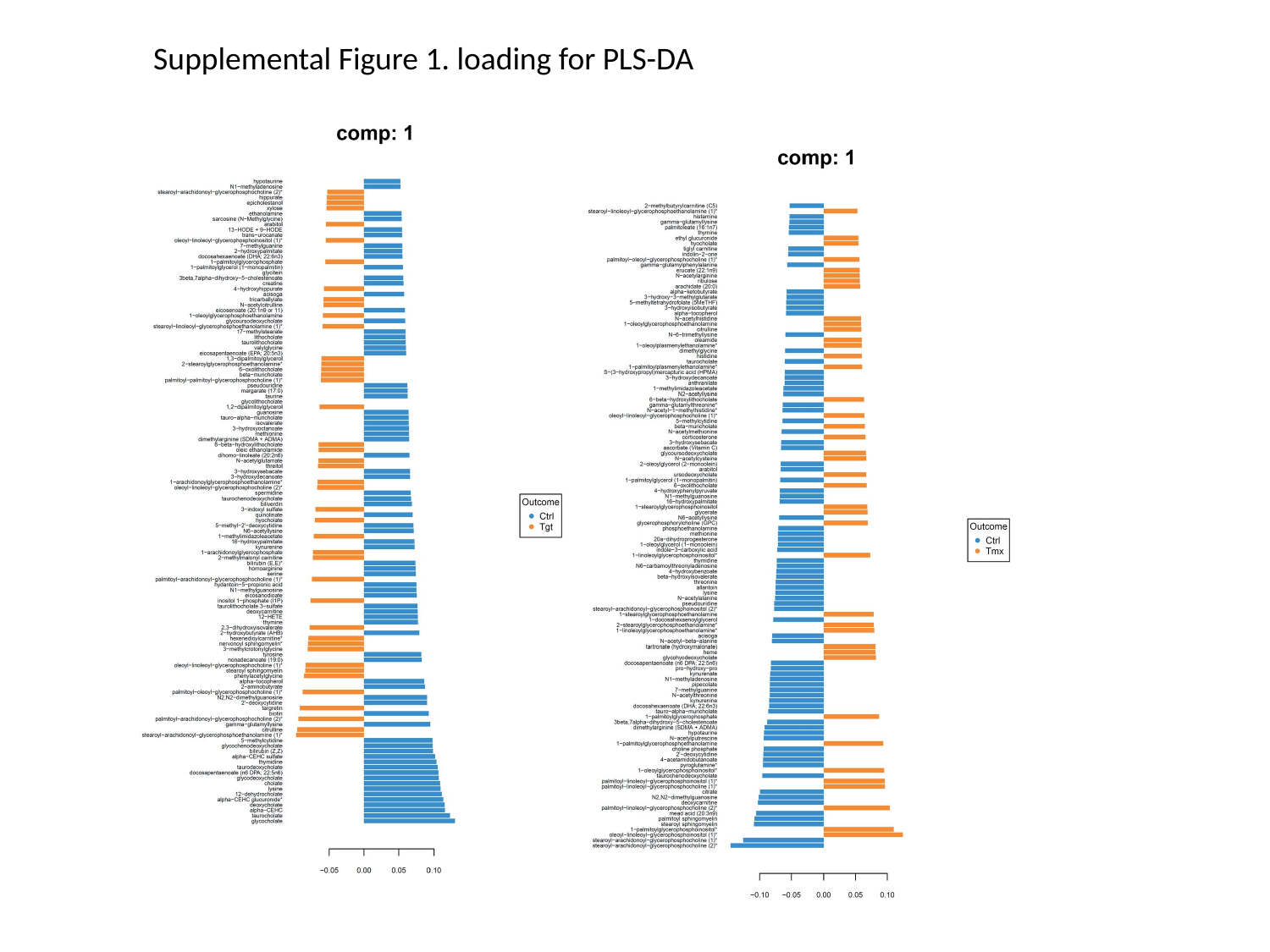

Supplemental Figure 1. loading for PLS-DA

## Slide 2
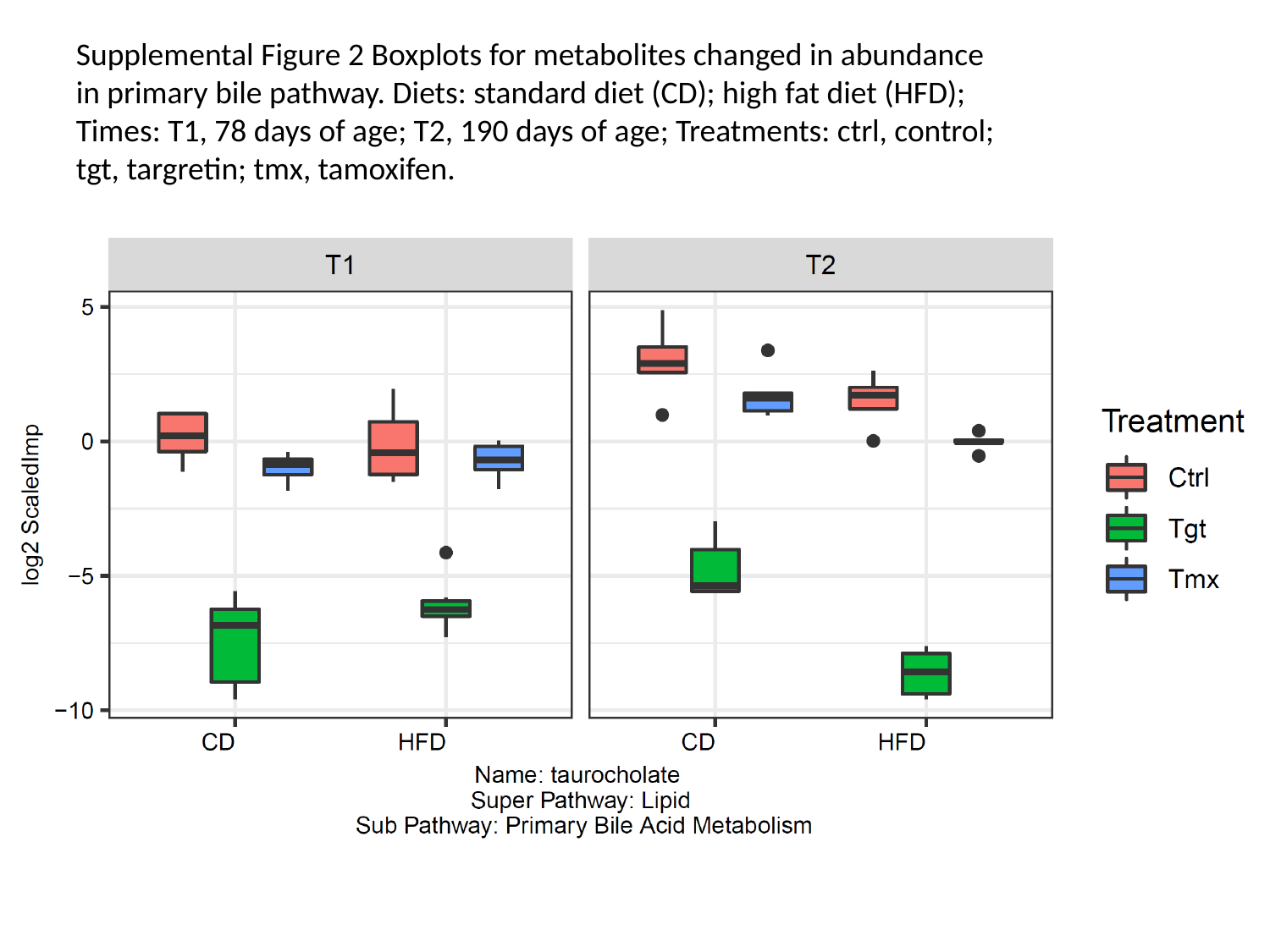

Supplemental Figure 2 Boxplots for metabolites changed in abundance in primary bile pathway. Diets: standard diet (CD); high fat diet (HFD); Times: T1, 78 days of age; T2, 190 days of age; Treatments: ctrl, control; tgt, targretin; tmx, tamoxifen.

## Slide 3
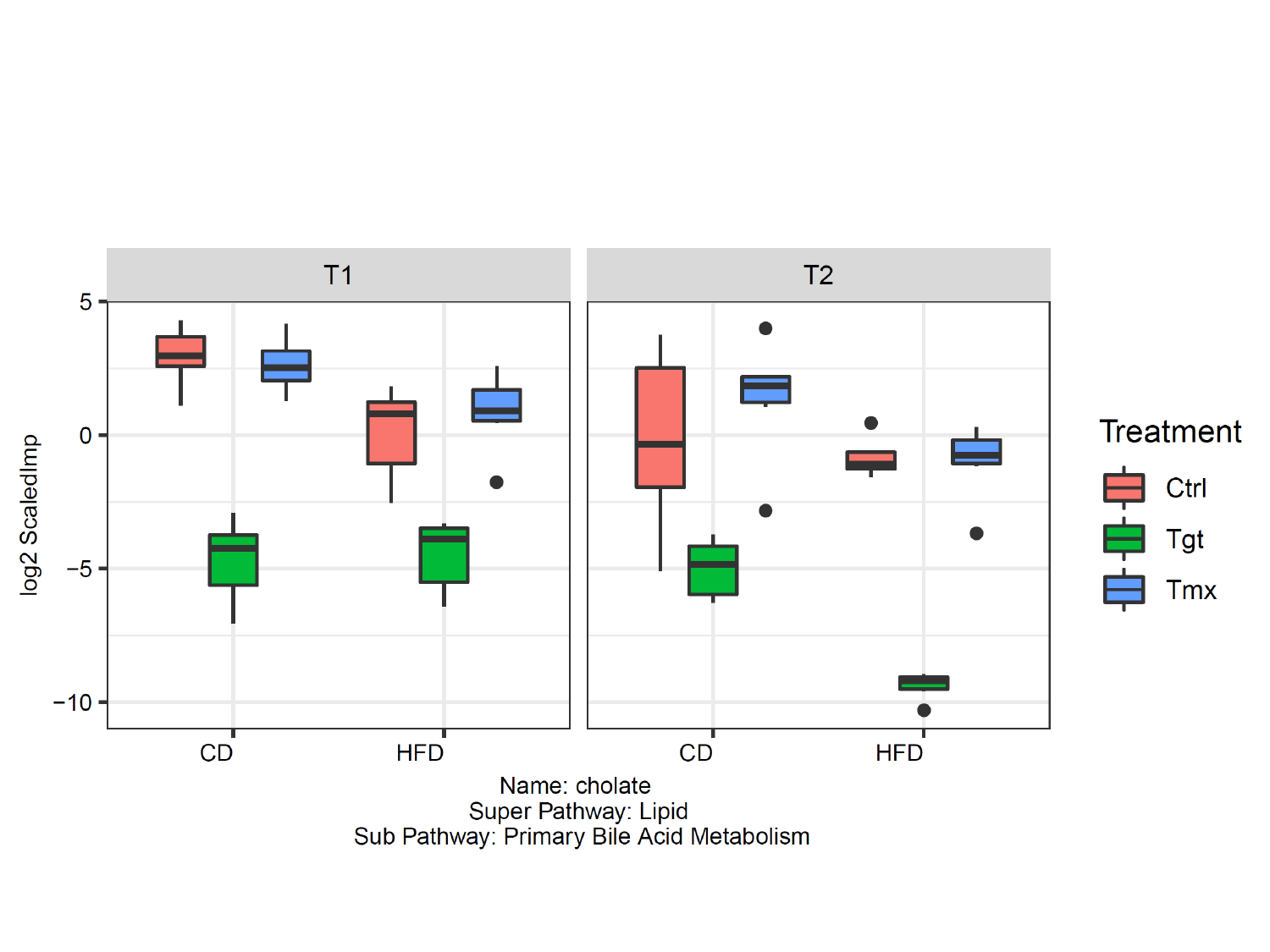

## Slide 4
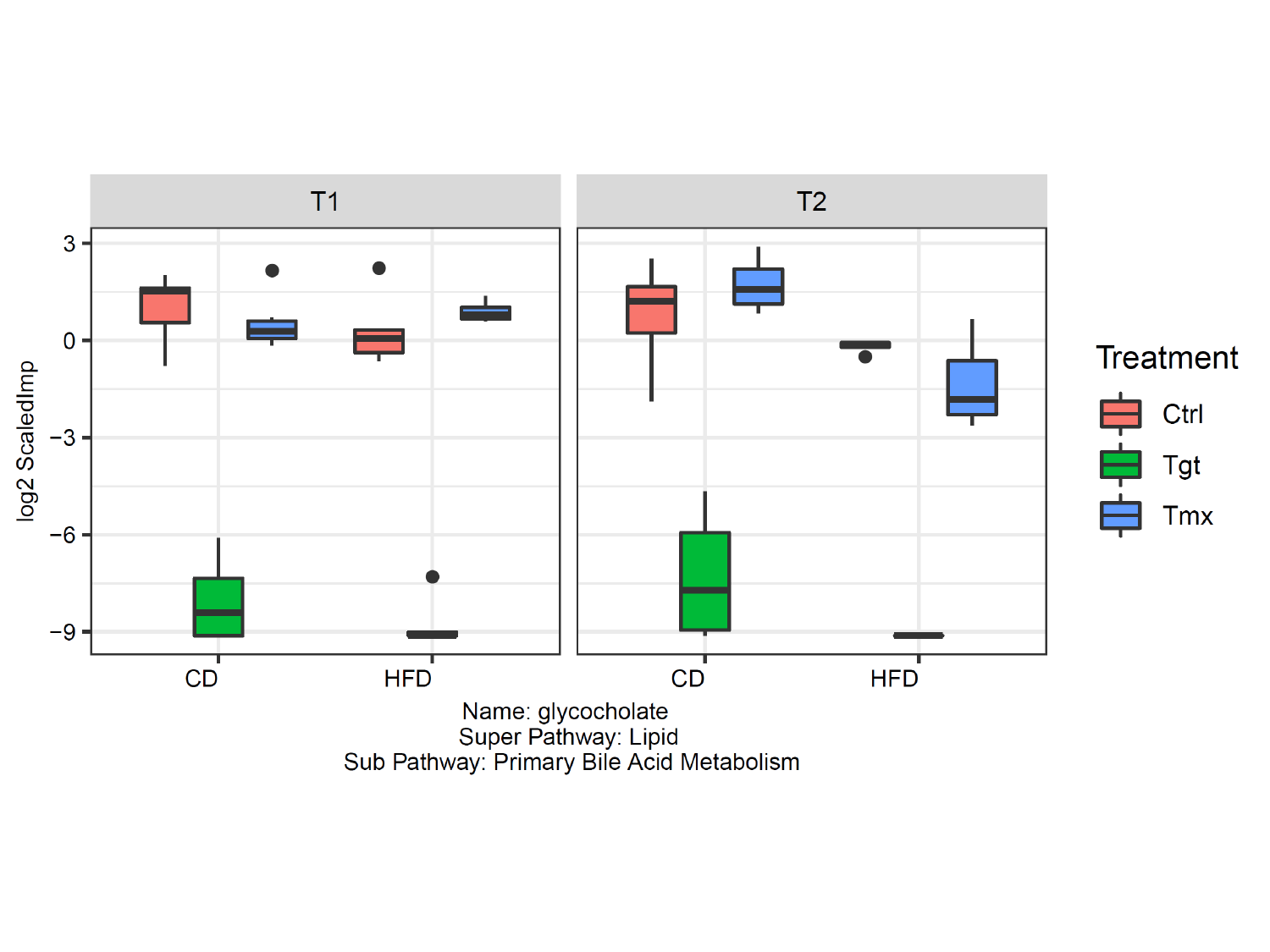

## Slide 5
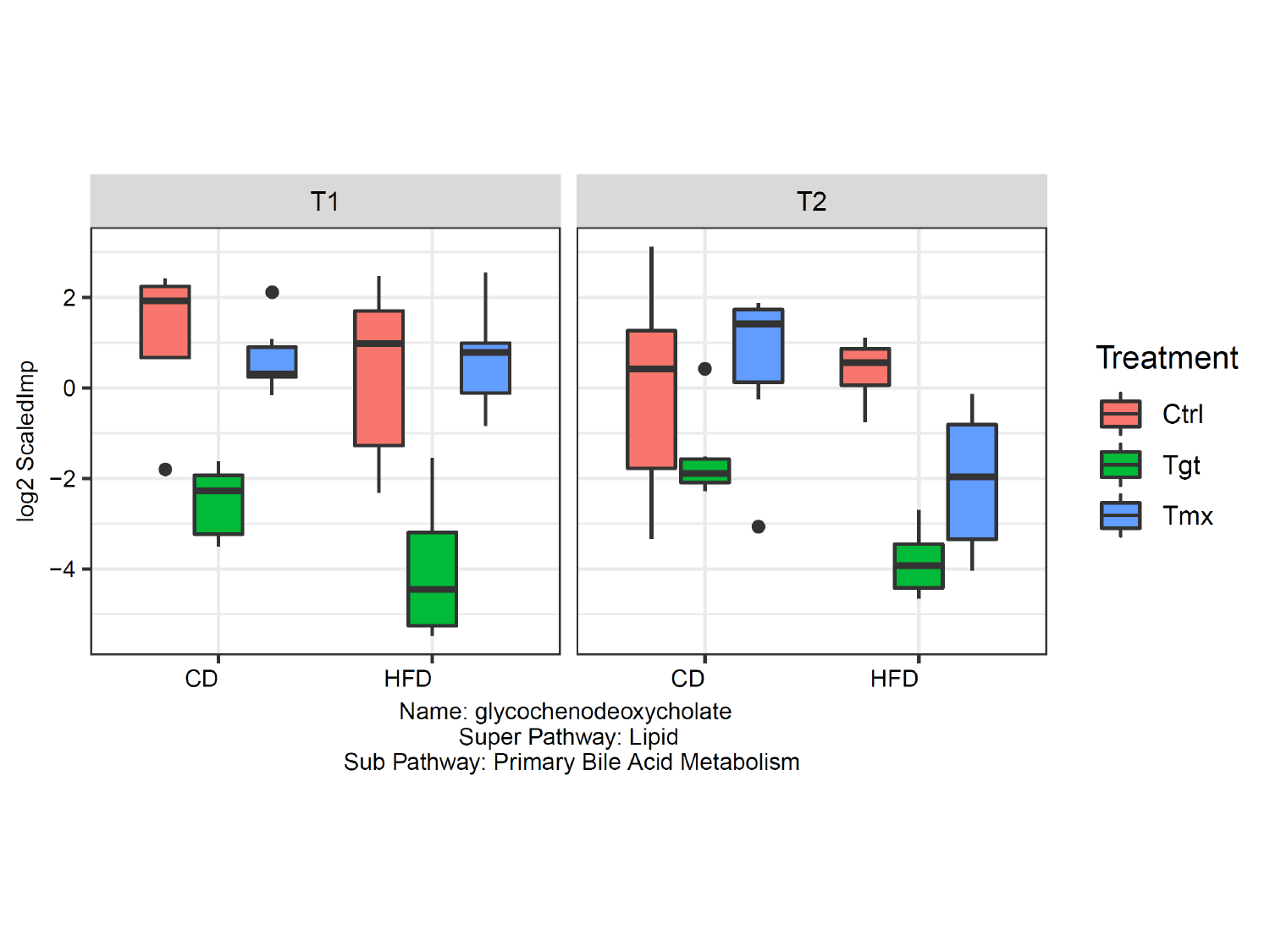

## Slide 6
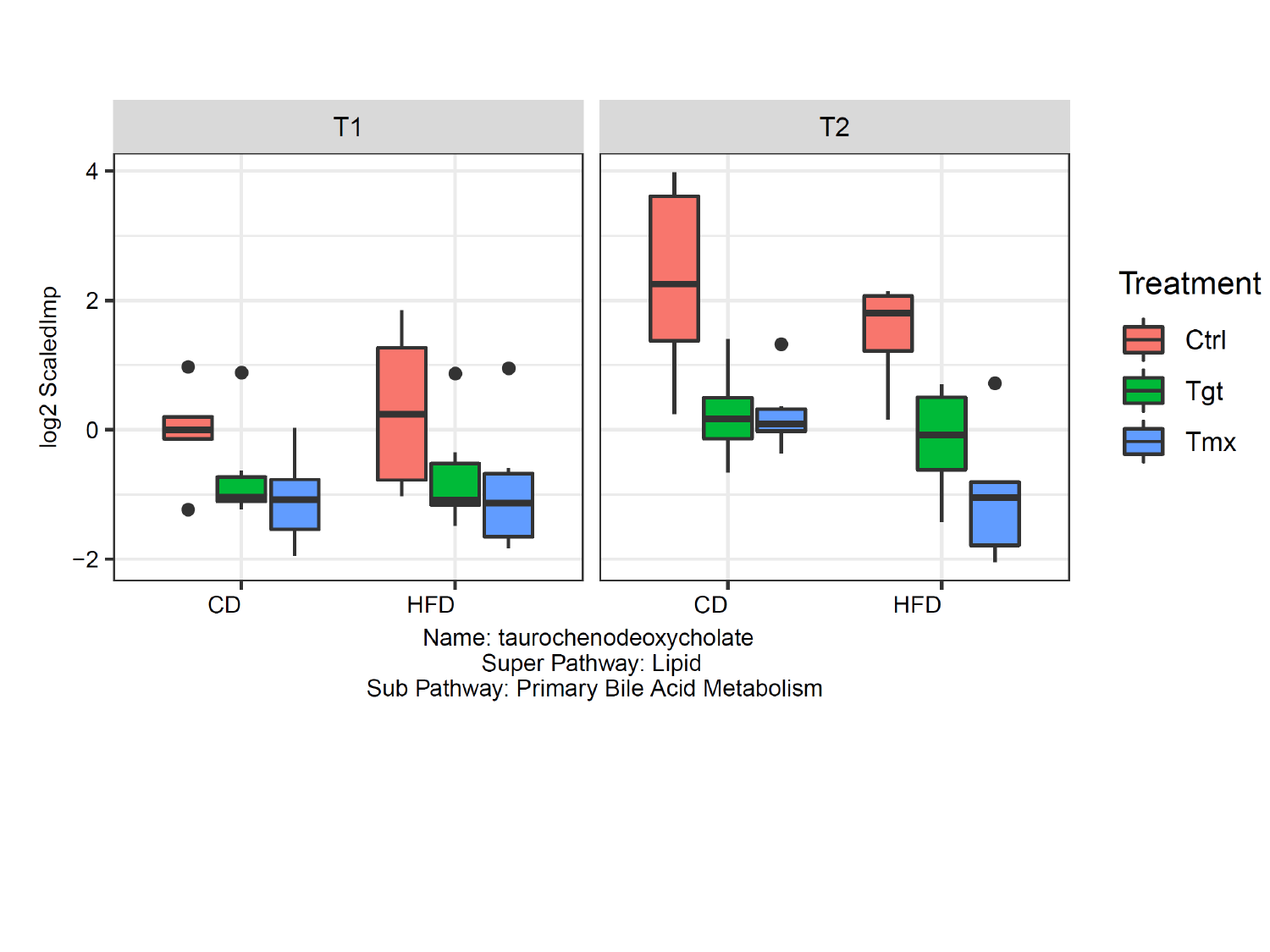

## Slide 7
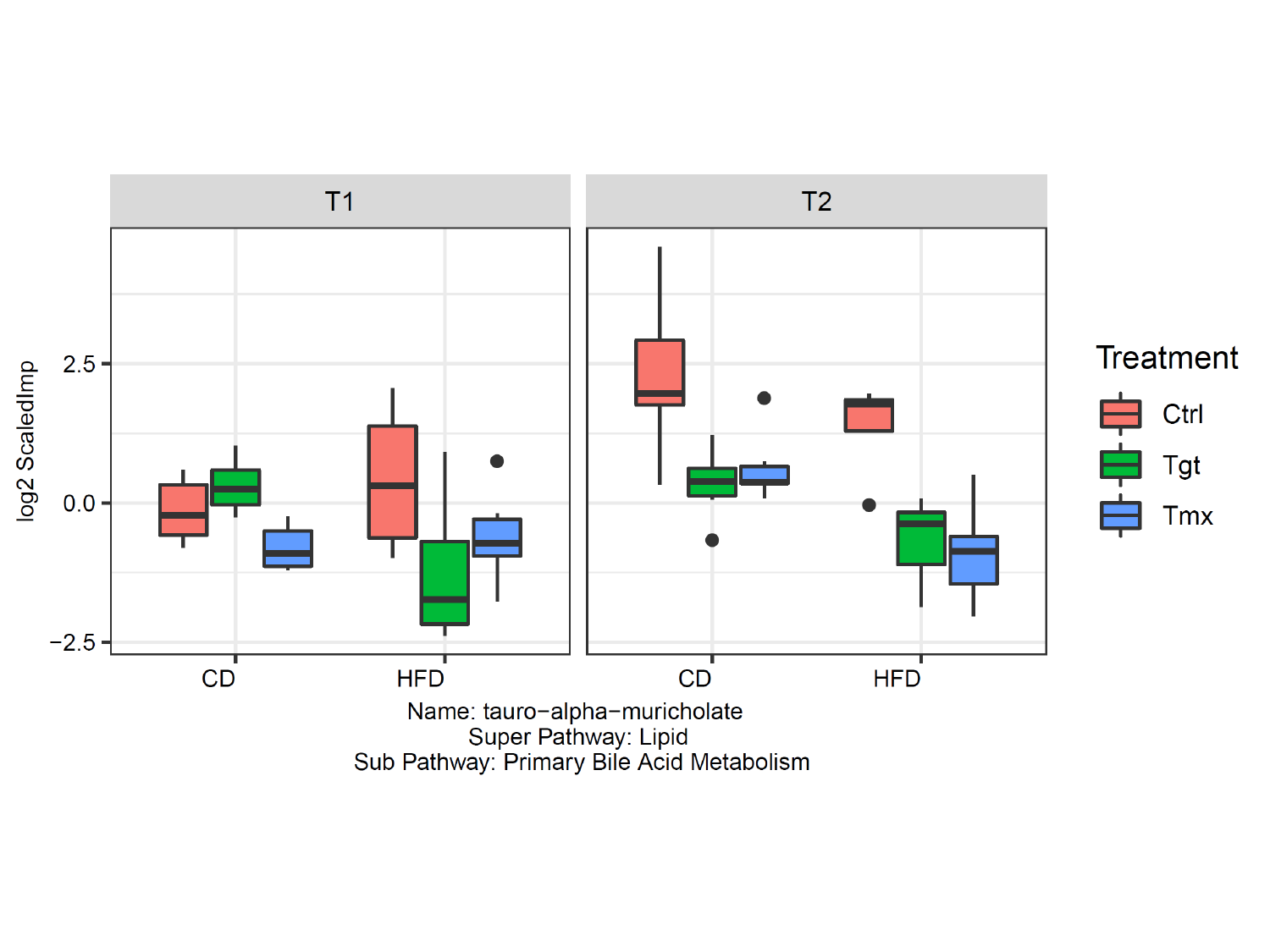

## Slide 8
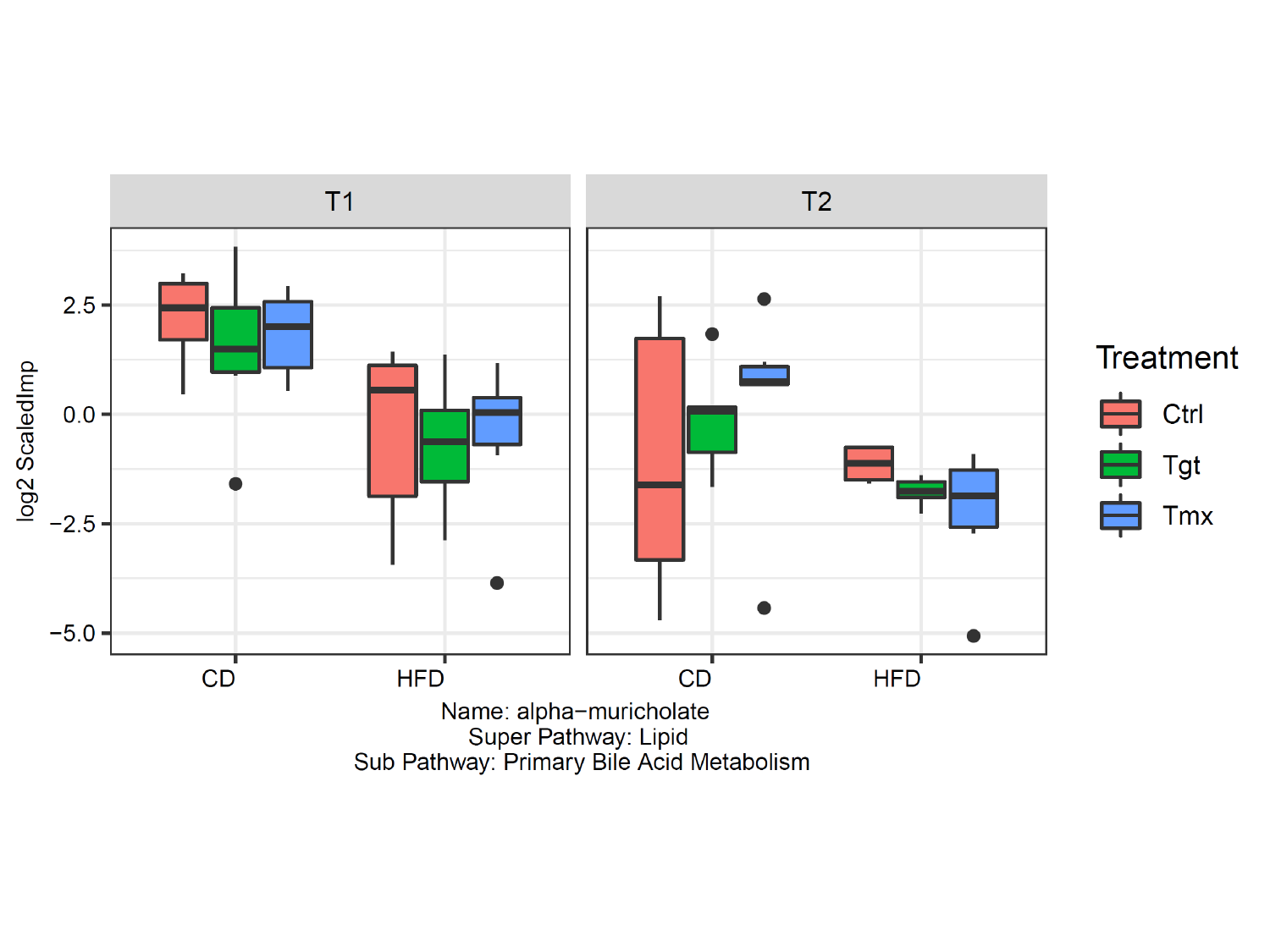

## Slide 9
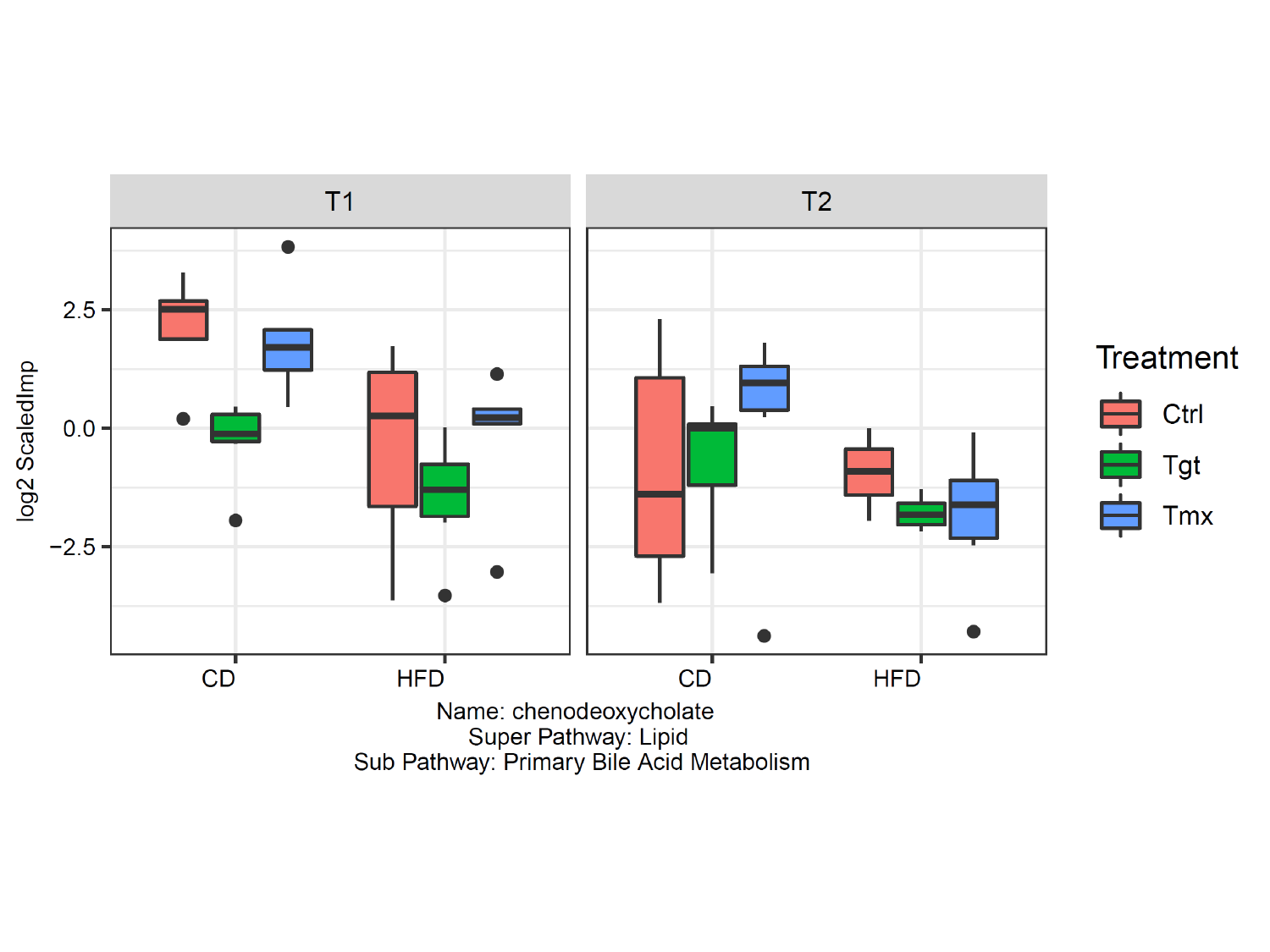

## Slide 10
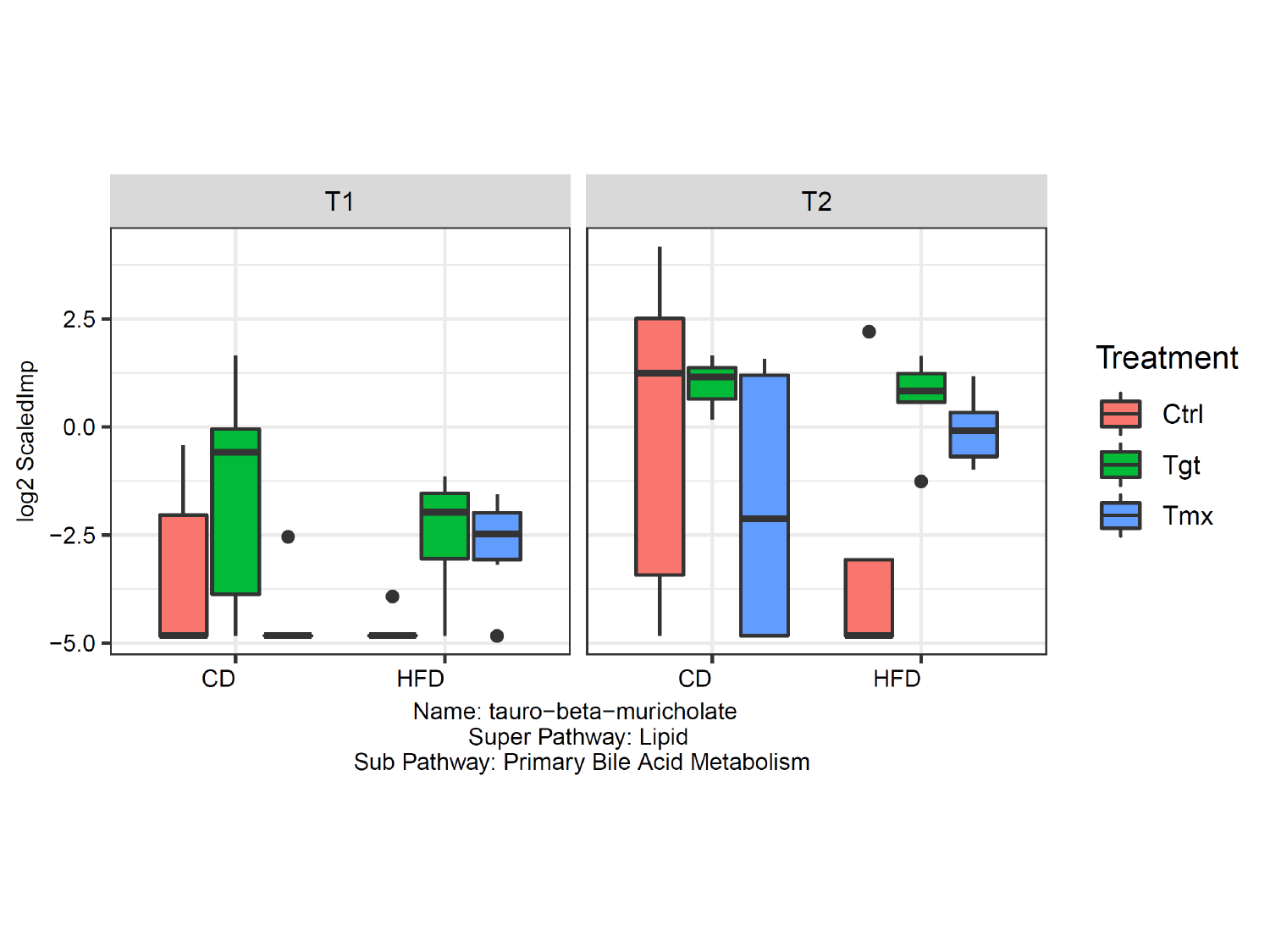

## Slide 11
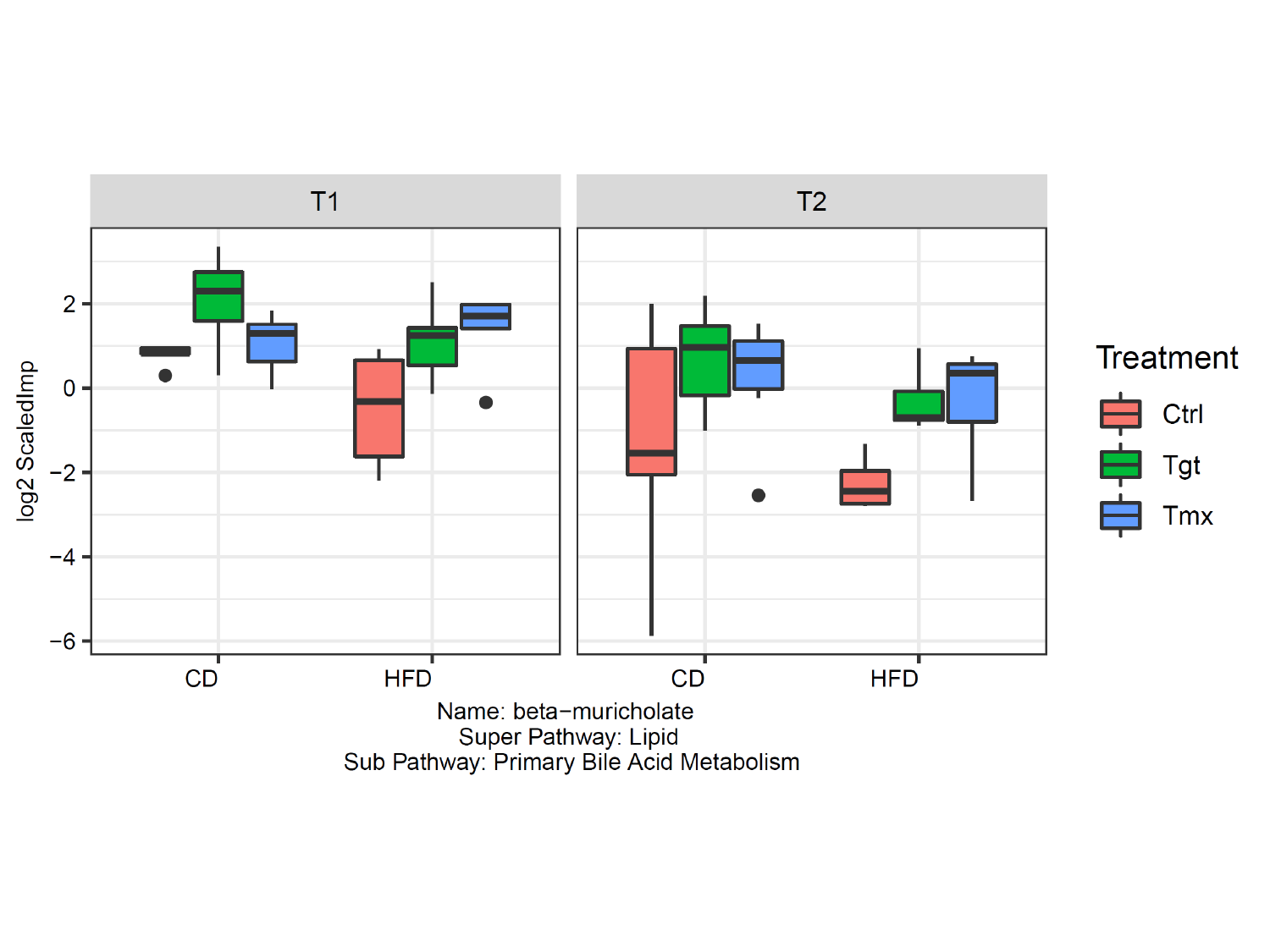

Supplement: Supplementary file 1 [file metabolites-09-00149-s001.zip › Supplemental figures_NCI PMx Paper_06222019.pptx]
